# B7-H3 inhibits apoptosis of gastric cancer cell by interacting with Fibronectin

**DOI:** 10.7150/jca.59263

**Published:** 2021-11-08

**Authors:** Meiyun Sun, Jinjing Xie, Dongze Zhang, Chunyang Chen, Simin Lin, Yan Chen, Guangbo Zhang

**Affiliations:** 1 Medical College of Soochow University, 199 Ren ai Road, Suzhou, Jiangsu Province, 215100, China.; 2 Jiangsu Institute of Clinical Immunology, The First Affiliated Hospital of Soochow University, 708 Ren min Road, Suzhou, Jiangsu Province, 215100, China.

**Keywords:** Gastric cancer, B7-H3, Fibronectin, adhesion, apoptosis

## Abstract

Anti-apoptosis has been widely accepted as a hallmark of malignancy. B7-H3, a type I transmembrane protein, plays a key role in anti-apoptosis and immune escape, but its regulation during cancer development remains unclear. To investigate how the effect of anti-apoptosis is regulated by B7-H3 in gastric cancer, we stably knocked down B7-H3 gene by shRNA in MGC-803 and MKN-45 cells. The correlation between B7-H3 and Fibronectin (FN) expression were investigated by bioinformatics in public data from TCGA (The Cancer Genome Atlas). Here, we reported that B7-H3 expression is positively correlated with FN in clinical gastric cancer samples, and B7-H3 promoted adhesion and inhibited apoptosis of gastric cancer cell through an FN-dependent pathway. Mechanistically, B7-H3 interacted with FN and subsequently activated PI3K/AKT signaling pathway, a critical mediator of oncogenic signaling. In addition, exogenous FN could inhibit the expression of pro-apoptosis-related proteins such as Caspase 3, Caspase 8, Caspase 9, Bax , p53, Apaf-1 and Cleaved PARP, and upregulated the levels of signal molecule p-PI3K, p-AKT and anti-apoptotic proteins Bcl-2 in B7-H3^high^ group, as compared with those in B7-H3^low^ group. In conclusion, we here for the first time revealed that B7-H3 inhibits apoptosis of gastric cancer cell through regulation of FN-mediated PI3K/AKT signaling pathways.

## Introduction

As one of most common malignant tumors, gastric cancer (GC) is presently mainly treated by surgery, chemotherapy, radiotherapy and immunotherapy, but these treatments still could not significantly improve patient's survival 1, and this also points to the need to explore the biological characteristics of gastric cancer.

B7-H3, which is a type I transmembrane protein of the B7 family, consist of two subtypes 2Ig B7-H3 and 4Ig B7-H3 2. B7-H3 was discovered in 2001 by Chapoval et al. 3, and reported as a co-stimulator molecule to enhance cytotoxic T lymphocyte (CTL) activity* in vitro* and stimulate the growth of CD4^+^ and CD8^+^ T cells^4^. Liu et al. found that B7-H3 inhibits the T cell response by promoting the secretion of the immunosuppressive cytokine IL-10 5. Moreover, B7-H3 affects T cells and other immune cells such as NK cells 6. In mouse model of liver cancer and EL-4 lymphoma, tumors expressing B7-H3 plasmid were significantly reduced, partly due to increased NK cell infiltration 7.

In addition, as a tumor-associated antigen, B7-H3 plays a key role in tumor progress. B7-H3 is high-expressed in gastric cancer, colorectal cancer, prostate cancer, kidney cancer, lung cancer, and breast cancer 4, 8, 9. Previous reports have shown that overexpression of B7-H3 contributes to immune evasion of tumors and promotes metastasis, thereby leading to a poor prognosis 10. In oral squamous cell carcinoma, overexpressed B7-H3 is positively associated with a more advanced clinical stage, larger tumor size, and lower survival 11. B7-H3 increases radiation resistance of gastric cancer cells by regulating autophagy and affecting apoptosis, cell cycle progression and DNA double-strand break repair 12. Also, B7-H3 activates the PI3K/AKT signal and up-regulates the protein level of Bcl-2, leading to the sustained growth of ovarian cancer 13. In renal clear cell carcinoma, the expressions of B7-H3 and tyrosine kinase receptor Tie-2 in tumor vessels are associated with disease progression and prognosis 14. In addition, inhibition of B7-H3 expression leads to inactivation of P38/MAPK signaling, thereby reducing cell proliferation and glycolysis of metastatic melanoma 15, 16. But due to the lack of corresponding molecule, the mechanism of action still remained unclear.

Fibronectin (FN) is a multifunctional glycoprotein present in the extracellular matrix (ECM) of plasma and tissues 17. FN exists in three forms, namely, cellular FN, plasma FN and fetal FN 18. FN has two disulfide bond-connected subunits with a molecular weight of 220-225 kD. Each subunit contains several ligand-binding domains that activate a series of FN-mediated signal transduction pathways to regulate biological functions, such as cell adhesion, migration, proliferation and embryonic development 17, 19.

Adhesion of metastatic cells plays a key role in tumor cell metastasis to host organ 20. Study showed that the adhesion ability of liver cancer HepG 2 cells decreases after knocking down the expression of B7-H3 20. We also found that adherent cells often show high-expressed B7-H3, while suspension cells hardly express B7-H3, which indirectly indicates that B7-H3 may promote cell adhesion.

Previous studies demonstrated that a high expression of B7-H3 or FN is related to a reduced tumor cell apoptosis, but the interaction between B7-H3 and FN in relation to cell apoptosis has not been investigated. In this study, we aimed to explore whether B7-H3 can regulate the apoptosis of gastric cancer cells through interaction with FN.

## Materials and methods

### Cell culture

MGC-803 and MKN-45 cell lines were purchased from the Cell Bank of the Chinese Academy of Sciences. DMEM high-glucose medium (#SH30243.01B, Hyclone, USA) containing 10% fetal bovine serum (FBS, #04-001-1ACS, Biological Industries, USA) and 1% Penicillin and Streptomyces (#C0222, Beyotime, China) was cultured in a saturated humidity incubator at 37℃ with 5% CO_2_.

### Cell transfection and infection

Silencing of B7-H3 sequences was designed by Shanghai GeneChem Co., Ltd., with hU6-MCS-Ubiquitin-EGFP-IRES-puromycin as a plasmid vector. The expression of B7-H3 in cells was knocked down with siRNA of the following target sequences: the sense sequence was 5'-GUGCUGGAGAAAGAUCAAATT-3', and the antisense sequence was 5'-UUUGAUCUUUCUCCACAGCACTT-3'. According to the instructions, MKN-45 cells and MGC-803 cells were divided into negative control (sh NC) and experimental group (sh B7-H3). 72 hours after the cell transfection, puromycin (#P8230, Solarbio, China) was added to the cells for two weeks, and then the transfection efficiency was detected by Western blot and qPCR.

### Co-immunoprecipitation (Co-IP) assays

MGC-803 cells and MKN-45 cells were lysed with IP lysis buffer (#P0013, Beyotime, China). Cell supernatant was collected by centrifugation and reacted with Ig G (#A7007, Beyotime, China) and Protein A+G Agarose (#P2055, Beyotime, China) following the instructions. Anti-B7-H3 antibody (#66481-1-Ig, Proteintech, China) and anti-FN antibody (#66042-1-Ig, Proteintech, China) were used for Western blot analysis.

### Cell adhesion analysis

The 96-well plate was coated with 20 μg/ml FN (#f81801, Solarbio, China), add with 2% BSA (#FMS-WB021, Fcmacs, China), and blocked for 1 hour. MGC-803 ctrl-sh NC, MGC-803 FN-sh NC, MGC-803ctrl-sh B7-H3, MGC-803 FN-sh B7-H3 cells were resuspended in 1% FBS medium and added to a 96-well plate, with each group having 5 replicate wells. After culturing for 1 hour, unadhered cells were removed, while the rest cells were fixed with 4% paraformaldehyde (#BL539A, Biosharp, China) and stained with crystal violet (#C0121, Beyotime, China). Photos were taken for statistical analysis. MKN-45 cell processing was conducted the same as MGC-803 cells.

### Apoptosis assays

The MGC-803 cells were inoculated into a 6-well plate and divided into the following groups: starvation + ctrl-sh NC (cells incubated with serum-free DMEM medium); starvation + FN ctrl-sh NC (cells incubated with serum-free DMEM medium and exogenous FN); starvation + ctrl-sh B7-H3 (cells incubated with serum-free DMEM); starvation + FN ctrl-sh B7-H3 (cells incubated with serum-free DMEM medium and exogenous FN). All the cells were cultured for 24 hours. The Annexin V-PE Apoptosis detect kit (#559763, BD, USA) was used to detect cell apoptosis. The processing of MKN-45 cells was conducted the same as that of MGC-803 cells.

### Immunohistochemistry staining

The tissues of 4 mice in the sh NC group and 4 mice in the sh B7-H3 group kept in the tissue fixator (#KSN00002, China) were dehydrated, embedded with paraffin, and then made into sections. After the antigen was extracted with 10 mM sodium citrate buffer (pH 6.0), the sections were incubated with anti-FN antibody (#66042-1-Ig, Proteintech, China). Following the incubation with the secondary antibody, the tissues were stained with DAB and counterstained with hematoxylin, dehydrated, mounted on the slides, and then the pictures were taken.

### Western blot analysis

The cultured MGC-803 and MKN-45 cells were collected, washed with PBS, and lysed on ice for 30 min with a RIPA lysis buffer (#P0013B, Beyotime, China) containing phosphatase inhibitors (#P1045, Beyotime, China) and protease inhibitors (#P1005, Beyotime, China). The protein concentrations of the cell lysates were determined by the BCA Protein Assay Kit (#P0012, Beyotime, China). Protein samples were separated on SDS-PAGE gel and transferred to polyvinylidene diflfluoride (PVDF) membranes (GE Healthcare, Germany), which were then labeled with the following primary antibodies overnight at 4 °C: anti-B7-H3 (#66481-1-Ig, Proteintech, China), anti-Fibronectin (#66042-1-Ig, Proteintech, China), anti-PI3K (#60225-1-Ig, Proteintech, China), anti-p-AKT (#66444-1-Ig, Proteintech, China), anti-AKT (#60203-2-Ig, Proteintech, China), anti-Bax (#5023,CST,USA), anti-Bcl-2 (#15071,CST,USA), anti-p-PI3K (#AF5905, Beyotime, China), anti-β-actin (#AF5001, Beyotime, China), anti-Caspase 3 (#AF1213 Beyotime, China), anti-Caspase 8 (#AF1243, Beyotime, China), anti-Caspase 9 (#AF1264, Beyotime, China), anti-Cytochrome C (#AF2047, Beyotime, China), anti-Apaf-1 (#AF1462, Beyotime, China), anti-Cleaved PARP (#AF1567, Beyotime, China) and anti-p53 (#AF1162, Beyotime, China). After TBST washing, the membranes were incubated with HRP-conjugated secondary antibodies (Beyotime, China), and the signals were detected by using an ECL detection kit (#36222ES60, Yeasen, China).

### RNA extraction and real-time quantitative PCR (qPCR)

The cells were collected for extracting total RNA using RNA-Quick Purification Kit (#RN001, Yishan, China). PrimeScript™ RT Master Mix Kit (#R0037A, Takara, Japan) and AceQ qPCR SYBR Green Master Mix (without ROX) kit (#q121, Vazyme, China) were employed to perform real-time quantitative PCR to detect the expression of B7-H3.

### Tumor growth *in vivo*

Five-week-old SPF BALB/C male nude mice were purchased from Shanghai Experimental Animal Center. All animal experiments were approved by the Institutional Animal Care and Use Committee of Soochow University (Suzhou, China). All the mice were randomly divided into groups. The sh B7-H3 and sh NC MGC-803 cells (5×10^6^) were suspended in 150μl PBS at a ratio of 1:1 and injected into the subcutaneous layer on the right side of the mice. The tumor volume was recorded every 5 days. After 5 weeks, the xenografts were harvested from the mice. Tumor tissues were immersed in tissue fixative for immunohistochemistry or placed at -80°C for Western blot. All the results of animal experiments were obtained blindly.

### Statistical analysis

The data were obtained from three independent experiment repetitions. Student's t-test was used for comparisons between two groups, and the results were expressed as the mean ± standard error (mean ± SEM). *P*<0.05 was considered as statistically significant.

## Results

### B7-H3 interacted with FN to promote adhesion of gastric cancer cells

**Figure 1:**
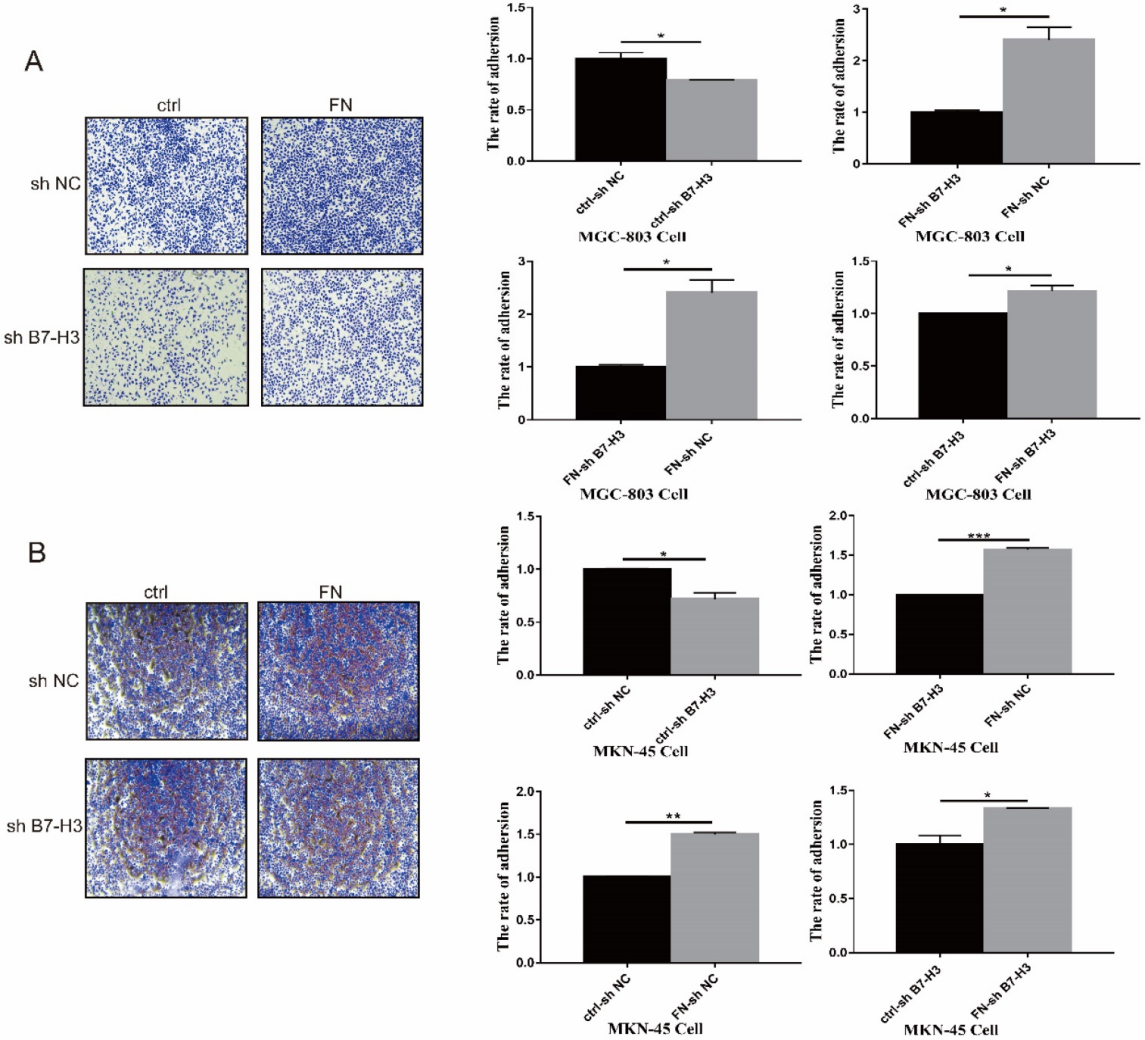
B7-H3 interacted with FN to promote cell adhesion. B7-H3 interacted with FN to promote the cell adhesion of MGC-803 cells (**A**) and MKN-45 cells (**B**). *p<0.05, **p<0.01, *** p<0.001.

MGC-803 sh B7-H3 cells and MKN-45 sh B7-H3 cells were successfully constructed for subsequent experiments (Fig. S1). In B7-H3^low^ group (MGC-803), the cell adhesion rate was significantly reduced when compared with that in B7-H3^high^ group (MGC-803). After exogenous FN treatment, the cell adhesion rate of B7-H3^high^ group (MGC-803) became greatly higher than B7-H3^low^ group (MGC-803), and the cell adhesion rate of B7-H3^low^ group (MGC-803) and B7-H3^high^ group (MGC-803) was increased noticeably with in addition of exogenous FN (Fig. 1A). The similar results were obtained in MKN-45 cells (Fig. 1B). Based on the above results, we found that in gastric cancer cells, B7-H3 bound to FN to promote cell adhesion.

### B7-H3/FN interaction inhibited gastric cancer cell apoptosis

**Figure 2:**
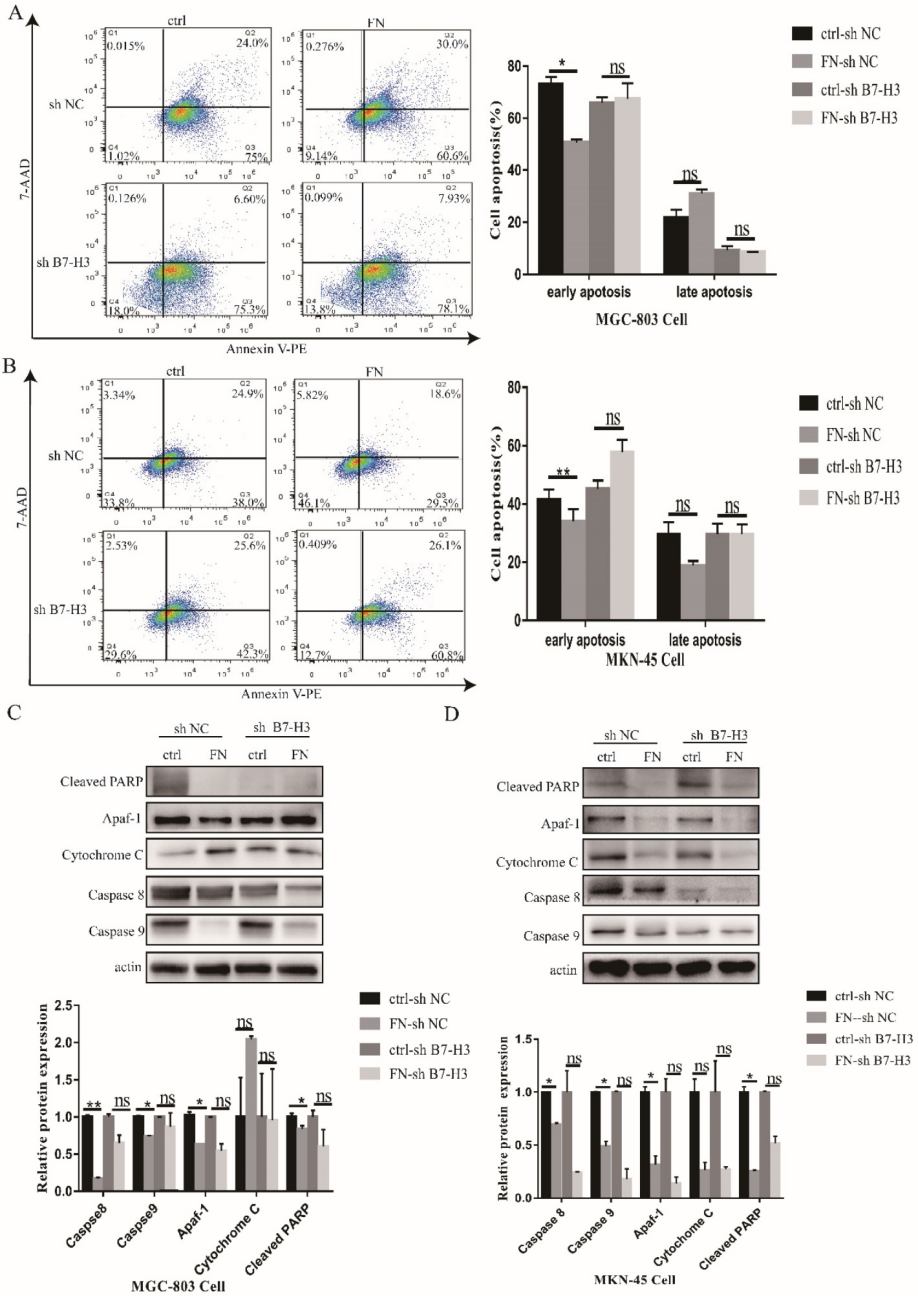
B7-H3/FN interaction inhibited the apoptosis of gastric cancer cells and down-regulated the expression of apoptotic protein. B7-H3/FN interaction inhibited the apoptosis of gastric cancer MGC-803 cells (**A**) and MNK-45 cells (**B**). Changes in the expressions of apoptotic proteins in MGC-803 cells (**C**) and MKN-45 cells (**D**). *p<0.05, **p<0.01, *** p<0.001.

As shown in Fig. 2A, early apoptosis of the cells in B7-H3^high^ group (MGC-803) was significantly reduced, but there was no significant change in the late apoptosis after exogenous FN treatment. In B7-H3^low^ group (MGC-803), there was no significant change in early or late apoptosis with the addition of exogenous FN. The similar results were also found in MKN-45 cells (Fig. 2B).

Subsequently, we examined the changes in apoptotic proteins. In MGC-803 cells, Cleaved PARP, Caspase 8 and Caspase 9 expressions were significantly decreased in the FN-sh NC group after exogenous FN stimulation compared with the ctrl-sh NC group(*P*<0.05),Cytochrome C expression increased(*P*<0.05), but apaf-1 expression had no significant change(*P*>0.05);Cleaved PARP and Cytochrome C expression were not significantly changed(*P*>0.05),Caspase 8 and Caspase 9 expression were significantly decreased(*P*<0.05) in the FN-sh B7-H3 group compared with the ctrl-sh B7-H3 group after exogenous FN stimulation, the expression of apaf-1 was increased(P<0.05), but the decrease of Caspase 9 expression in ctrl-sh B7-H3 group was less than that in ctrl-sh NC group after adding exogenous FN(Fig. 2C),and similar results were obtained in MKN-45 cells (Fig. 2D).These results suggest that exogenous FN and B7-H3 may inhibit the apoptosis of gastric cancer cells through Caspase 9.

### The interaction between B7-H3 and FN in gastric cancer cells

**Figure 3:**
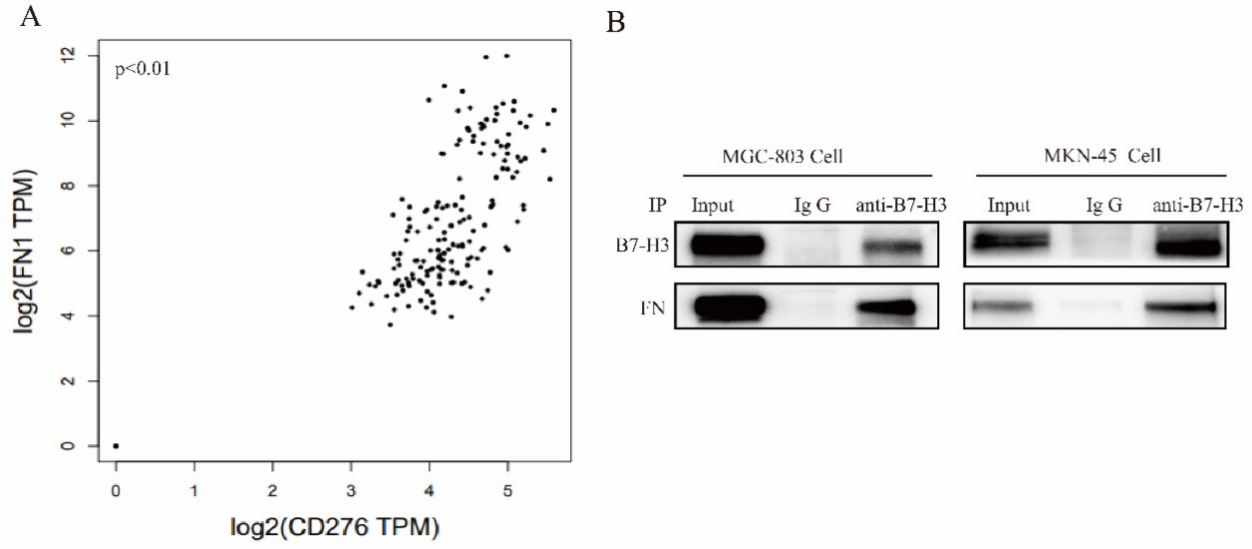
** Detection of the interaction between B7-H3 and FN.** (**A**)The correlation coefficients of B7-H3 and FN genes were analyzed by TCGA database; (**B**) Co-IP assay were used to detect the interaction between B7-H3 and FN. *p<0.05, **p<0.01, ***p<0.001.

Correlation between B7-H3 and FN in gastric cancer was identified from the TCGA database (Fig. 3A). We found that FN polyclonal antibodies (#AF1918, R&D, USA) can recognize FN and B7-H3 (data not shown), suggesting that FN and B7-H3 may interact with each other through the formation of natural complexes.

FN was co-precipitated by B7-H3 in MGC-803 and MKN-45 cells, and this further confirmed their interaction between B7-H3 and FN (Fig. 3B). Based on the above results, it can be found that B7-H3 and FN bind to each other in MGC-803 and MKN-45 cells.

### B7-H3/FN interaction can activate PI3K/AKT signaling pathway and inhibit the apoptosis of gastric cancer cells

**Figure 4:**
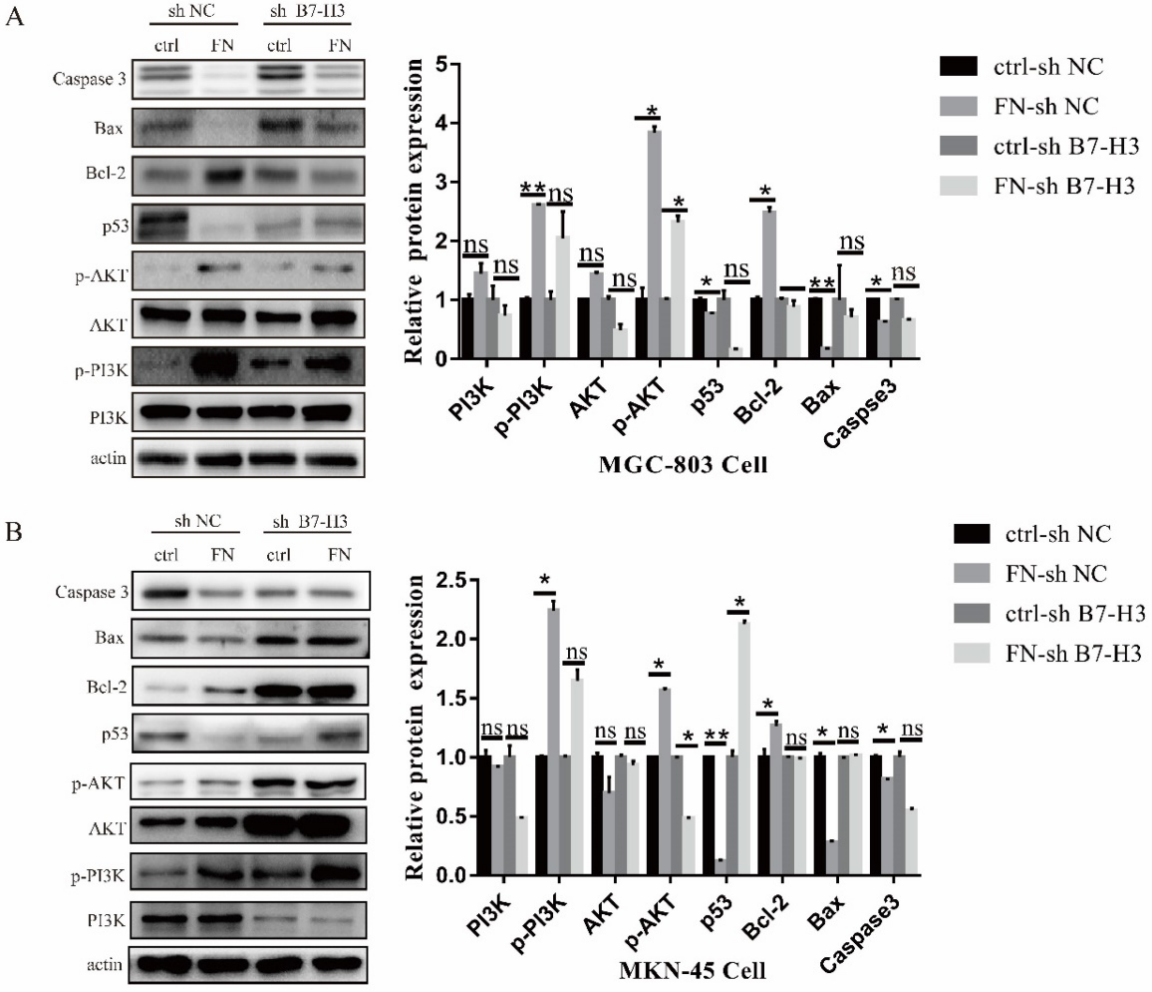
** B7-H3/FN interaction may inhibit the apoptosis of gastric cancer cells by activating the PI3K/AKT signaling pathway.** B7-H3/FN interaction may activate the expression of PI3K/AKT signaling pathway in MGC-803 cells (**A**) and MKN-45 cells (**B**). *p<0.05, **p<0.01, *** p<0.001.

In MGC-803 cells, the expressions of signal molecular p-PI3K, p-AKT and anti-apoptotic protein Bcl-2 were significantly up-regulated, whereas the expression levels of pro-apoptotic protein p53, Bax and Caspase 3 were sharply down-regulated after adding exogenous FN in ctrl-sh NC group. In ctrl-sh B7-H3 group, the expression levels of these protein molecules did not change with the addition of exogenous FN (Fig. 4A), and the similar results were obtained in MKN-45 cells (Fig. 4B). These experimental results indicated that B7-H3/FN interaction can inhibit the apoptosis of gastric cancer cells by activating the PI3K/AKT signaling pathway.

### Silencing the expression of B7-H3 could inhibit tumor growth *in vivo*

**Figure 5:**
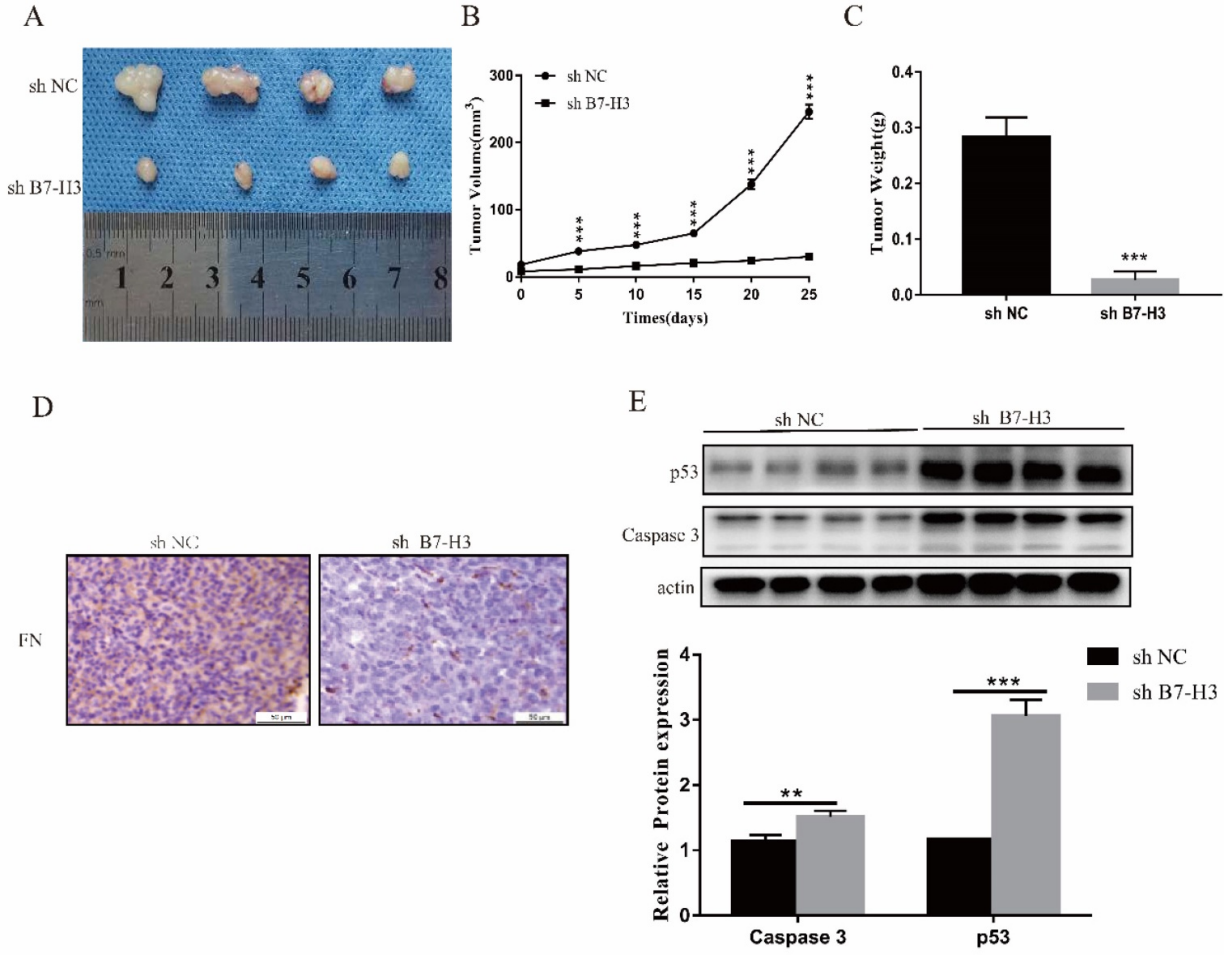
** B7-H3 could inhibit growth of tumor *in vivo*.** The size, volume and weight of the tumor after silencing the expression of B7-H3 (**A-C**). Immunohistochemistry to detect the levels of FN in mouse tumors. Scale bar, 50 µm (**D**). Western blot was used to detect the expressions of p53 and Caspase 3 in mouse tumors(E). *p<0.05, **p<0.01, ***p<0.001.

Due to the short action time of FN, only sh NC and sh B7-H3 cells of MGC-803 cells were used to establish mouse xenograft tumor models. We found that in sh B7-H3 group, the tumor size, volume and weight were all reduced to varying degrees (Fig. 5A-C). Subsequently, it was also found that compared with the sh NC group, the FN level in the sh B7-H3 group was decreased (Fig. 5D), and this showed that the adsorbed FN level was decreased, suggesting that the binding of B7-H3 and FN may be caused by sedimentation. At the same time, we also detected the expressions of apoptotic proteins p53 and Caspase 3, and observed that in sh B7-H3 group, the expressions of p53 and Caspase 3 were significantly up-regulated (Figure 5E). Our results showed that silencing the expression of B7-H3 promoted the expression of apoptotic proteins.

## Discussion

As an immunomodulatory factor, the role of B7-H3 varies in different types of human tumors, such as gastric cancer, kidney cancer, and colorectal cancer 21. B7-H3 regulates cancer-related signaling pathways in the non-immune system to affect tumor progression 6, 20. Chen et al. found that hB7-H3 molecule has an effect on the adhesion of extracellular matrix, such as FN, collagenⅣ and laminin, and its mediated adhesion can effectively promote the adhesion of tumor cells, but the direct binding of B7-H3 molecule to FN and its functional mechanism was not confirmed 22. In this study, we preliminarily explored the regulatory relationship of the interaction between B7-H3 and FN on cell adhesion. Our experimental results showed that B7-H3 interacted with FN, and that the interaction of B7-H3 and FN could effectively promote MGC-803 and MKN-45 cell adhesion. Previous studies demonstrated that cell adhesion can affect tumor cell proliferation, migration, apoptosis and other processes, and that cell adhesion to extracellular matrix such as FN may reduce its resistance to chemical drugs 23-26.

Apoptosis is an active and orderly cell death regulated by genes. In the presence of ATP or dATP, Cytochrome C will combine with Apaf-1 to form a complex that activates Caspase-9, thereby activating Caspase-3 and cleaving the substrate to induce apoptosis 27. Bcl-2 prevents apoptosis by preventing pro-apoptotic molecules from entering the cytoplasm from mitochondria, while Bax promotes apoptosis by inducing mitochondrial outer membrane permeability 28-30. The ratio of Bax/Bcl-2 determines the sensitivity of cells to death signal 31. Apaf-1 plays an important role in the mitochondrial apoptotic pathway, because it enhances a variety of processes including the formation of apoptotic bodies with Cytochrome C and dATP, and can activate Caspase-3 and induce cell apoptosis 32. Caspase 8 is a key mediator of adhesion cell death 33. In our present study, we found that in sh NC group, exogenous FN significantly inhibited cell apoptosis, while in sh B7-H3 group, apoptosis did not show great change after the addition of exogenous FN. In addition, we found that in sh NC group, exogenous FN sharply down-regulated the expressions of pro-apoptotic proteins Caspase 8, Caspase 9, Apaf-1 and Cleaved PARP, while in sh B7-H3 group, the expressions of pro-apoptotic proteins Caspase 8, Caspase 9, apaf-1 and Cleaved PARP did not change greatly after addition of exogenous FN. These data suggested that B7-H3 inhibited apoptosis of gastric cancer cells through interacting with FN.

More evidence showed that extracellular matrix adhesion activates the PI3K/AKT signaling pathway, thereby inhibiting various forms of tumor cell death 34, 35. PI3K is a key regulator of the formation of adhesion junction, and it can directly bind to E-Cadherin, thereby regulating adhesion-mediated cell cycle progression 36, 37. Studies have also shown that down-regulation of AKT protein expression can significantly induce cell apoptosis and inhibit cell proliferation 38. It has been determined that the downstream target proteins of the AKT signaling pathway are protein molecules related to apoptosis, such as Bax, Bcl-2 and Caspase 3 39. Activated AKT may affect cell cycle progression and tumor growth via the p53 pathway and Bcl-2 protein family 40. In this research, we found that in sh NC group, the expressions of p-PI3K, p-AKT and Bcl-2 were up-regulated, and the expressions of p53, Bax and Caspase 3 were down-regulated following the treatment of exogenous FN. However, in sh B7-H3 group, the expression of these proteins did not change significantly after the addition of exogenous FN. Thus, B7-H3 inhibited the apoptosis of gastric cancer cells and activated PI3K/AKT signaling pathway through interacting with FN.

*In vivo* studies indicated that B7-H3 plays an important role in tumor progression. Previous report also proved that FN has a certain correlation with cell proliferation 41. Our experimental results showed that low-expressed B7-H3 inhibited the growth of mouse tumors, and that the level of FN was also reduced. At the same time, the expressions of p53 and Caspase 3 were up-regulated.

In conclusion, we found that B7-H3 inhibited the apoptosis of gastric cancer cell through interacting with FN, and that the related signaling pathway may be PI3K/AKT. These experimental results indicated that adhesion and PI3K/AKT signaling pathway is possibly correlated, and may be a potential target for tumor therapy in the future.

## Supplementary Material

Supplementary figure.
