# Supplementary material for: B7-H3 inhibits apoptosis of gastric cancer cell by interacting with Fibronectin
Source: J Cancer. 2021 Nov 8;12(24):7518–26. doi: 10.7150/jca.59263 (PMC8734419; doi:10.7150/jca.59263)
Supplement: Supplementary file 1 — Supplementary figure. [file jcav12p7518s1.pdf]

Supplementary Fig. 1:

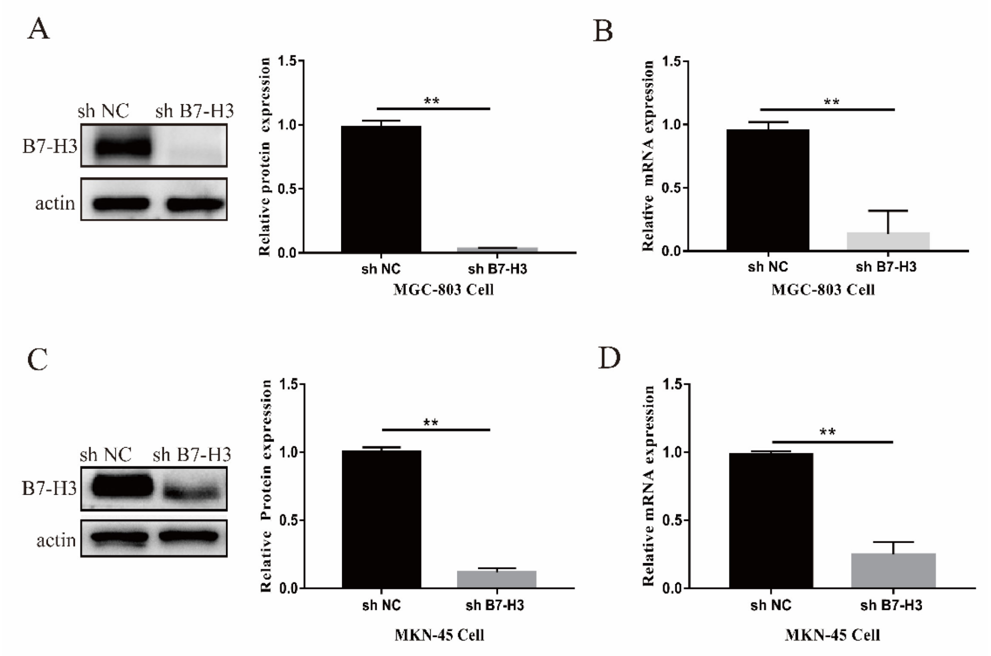

Supplementary Fig. 1: Construction and identification of cell lines. Construction and identification of MGC-803 cell line(A-B) and MKN-45 cell lines(C-D).
